# Deep learning for causal inference using low birth weight in midwife-led continuity care intervention in north Shoa zone, Ethiopia

**DOI:** 10.3389/frai.2025.1484299

**Published:** 2025-09-24

**Authors:** Wudneh Ketema Moges, Awoke Seyoum Tegegne, Aweke A. Mitku, Esubalew Tesfahun, Solomon Hailemeskel

**Affiliations:** ^1^Department of Statistics, College of Science, Bahir Dar University, Bahir Dar, Ethiopia; ^2^Department of Statistics, College of Science, Debre Berhan University, Debre Berhan, Ethiopia; ^3^Department of Data Science, College of Computing, Debre Berhan University, Debre Berhan, Ethiopia; ^4^Global Change Institute (GCI), Faculty of Science, University of the Witwatersrand, Johannesburg, South Africa; ^5^Department of Public Health, College of Health Science, Debre Berhan University, Debre Berhan, Ethiopia; ^6^Department of Midwifery, College of Health Science, Debre Berhan University, Debre Berhan, Ethiopia

**Keywords:** causal deep learning, low birth weight, precision in estimating heterogeneous treatment effects, average treatment effect, midwife-led continuity care

## Abstract

**Introduction:**

Low birth weight (LBW), under 2,500 g, poses health risks, though not always requiring treatment. Early detection of high-risk pregnancies enables preventive care, improving outcomes for mother and baby. This study aimed to establish cause-and-effect relationships using Causal Deep Learning (CDL) models that reduce bias and estimate heterogeneous treatment effects on LBW in the Midwife-Led Continuity Care (MLCC) intervention.

**Methods:**

This study used a quasi-experimental study design (August 2019–September 2020) in North Shoa, Ethiopia, and enrolled 1,166 women divided into two groups: one receiving MLCC and the other receiving other professional groups for comprehensive antenatal/postnatal care. The dataset and code are provided in data availability section. Our model combines counterfactual convolutional neural networks to analyze time-based patterns and Bayesian Ridge regression to reduce bias in propensity scores. We use Counterfactual Regression with Wasserstein Distance (CFR-WASS) and Counterfactual Regression with Maximum Mean Discrepancy (CFR-MMD) to balance patient characteristics and improve counterfactual estimates of treatment effects. This approach strengthens causal insights into how MLCC interventions affect LBW outcomes.

**Result:**

The Deep neural networks (DNN) model showed strong predictive accuracy for LBW, with 81.3% training and 81.4% testing performance, an area under the curve (AUC) of 0.88, enabling the reliable early identification of high-risk pregnancies. The study found a strong link between meconium aspiration syndrome (MAS) and LBW (*p* = 0.002), but this does not mean MAS directly causes LBW. MAS likely results from fetal distress or other pregnancy complications that may independently affect LBW. While statistical associations exist, clinical causation remains unproven; therefore, the counterfactual analysis showed MLCC could help reduce LBW risk. CFR-WASS achieved high accuracy (84%) while the precision in heterogeneous treatment effect (PEHE = 1.006) and the average treatment effect (ATE = 0.24), and CFR-MMD PEHE of 1.02, ATE of 0.45, demonstrating potential for tailored treatment strategies. DNN and multilayer perceptrons uniquely identified key neural weights and biases favoring normal birth weight while suppressing LBW predictions, offering interpretable insights for clinical risk assessment.

**Conclusion:**

The CFR-WASS/CFR-MMD model strengthens LBW prediction by identifying crucial factors like MAS and healthcare access, while accurate PEHE and ATE estimates support data-driven prenatal care and targeted interventions for healthier outcomes.

## Introduction

Low birth weight (LBW), defined as a birth weight below 2,500 grams, is a major global public health issue linked to higher neonatal mortality and long-term adverse health outcomes (Desta, 2019; Endalamaw et al., 2018). Despite existing interventions aimed at reducing LBW, there remains a critical gap in understanding how these strategies differentially affect subpopulations of pregnant women, particularly in low-resource settings (Park et al., 2020; Koivu et al., 2023).

Antenatal care (ANC) has been widely recognized as a key factor in reducing LBW risk. Studies consistently show that inadequate ANC, often defined as fewer than four visits during pregnancy, is strongly associated with higher LBW incidence. For example, (Roslina, 2020) found that mothers receiving fewer than four ANC visits had a 1.9 times greater risk of delivering LBW infants compared to those with adequate care. Similarly, (Sunarni et al., 2018) demonstrated that regular ANC attendance significantly improves birth weight outcomes, reinforcing the need for targeted maternal healthcare policies.

This gap relates to the concept of heterogeneous treatment effects. Heterogeneous treatment effects occur when the effectiveness of an intervention varies across different groups of individuals (Kent et al., 2018). In the context of LBW prevention, reduction-specific Midwife-Led Continuity Care (MLCC) is highly effective for one sub-group of pregnant women. It has a minimal or negative effect on another subgroup (Sandall et al., 2024). Unfortunately, most studies on LBW prevention have focused on average treatment effects (ATE) and neglected the potential for heterogeneous treatment effects. This limitation hinders the development of precisely targeted interventions to conduct the current study, which contributes to filling a critical knowledge or awareness gap regarding the adverse treatment effect.

In healthcare research, observational studies estimate causal effects, specifically the impact of MLCCs or interventions on medical outcomes. However, quasi-experimental studies are the gold standard for establishing cause-and-effect relationships, and practical constraints such as ethical, logistical, or financial limitations may make them infeasible. In such situations, researchers rely on quasi-experimental studies to explore causal effects. These studies address potential bias by considering confounders, although both approaches rely on untestable assumptions (Zawadzki et al., 2023). Deep learning (DL) is a subset of machine learning (ML) that uses deep neural networks (DNNs) to recognize patterns in large, complex datasets, achieving state-of-the-art results in fields such as computer vision and health sciences (Keles and Bagci, 2023). In neonatology, DL has proven revolutionary, particularly in tasks such as survival analysis, neuroimaging, and diagnosis of conditions such as retinopathy of prematurity (Keles and Bagci, 2023). Furthermore, DL models are complex networks that learn independently without human intervention. These models have multiple layers, enabling them to process information without explicit human guidance (Taye, 2023).

Deep learning is preferred over the existing methods for causal inference in LBW due to its ability to model complex and non-linear relationships. DL models, such as neural networks, can capture intricate interactions and confounding factors, providing more accurate causal effect estimates (Fan et al., 2023). Frameworks such as Counterfactual Regression with Wasserstein Distance (CFR-WASS) and Counterfactual Regression with Maximum Mean Discrepancy (CFR-MMD) integrate causal inference techniques, enhancing robustness and generalizability (Meinshausen, 2018; Shalit et al., 2017). While the existing methods are somehow more interpretable, DL’s superior predictive performance and flexibility make it a powerful tool for identifying causal factors in LBW studies, going beyond prediction to uncover actionable insights (Uauy et al., 2013).

Recent research highlights the integration of causal inference with DL to enhance model robustness, interpretability, and generalizability. This approach addresses limitations in existing DL, which may capture spurious correlations and lack interpretability (Jiao et al., 2024). By integrating causal effects into DL models, analysts have enhanced predictive performance and the interpretability of results in complex domains such as electronic health records (Ghosh et al., 2018). The combination of ML and causal inference methods has also shown promise in system dynamics modeling, enabling better forecasting and understanding of complex interactions across various disciplines (Koch et al., 2025). To facilitate adoption, researchers have developed frameworks and tutorials for implementing DL-based causal inference methods, focusing on observational causal estimation and extending causal inference to settings with non-linear confounding and diverse data types (Koch et al., 2025). This emerging field offers significant potential for advancing our understanding of cause-and-effect relationships in complex systems. Therefore, DL models augmented with causal inference techniques are better equipped to address confounding variables and biases, resulting in more precise and dependable predictions. This approach helps to detect targeted interventions to prevent LBW, leading to better health outcomes for mothers and babies, and it also helps to connect causal inference and DL within quasi-experimental settings (Zhang et al., 2022; Samek et al., 2021). Causal Deep Learning (CDL) leverages partial causal knowledge among some and not necessarily all variables of interest and quantitatively characterizes the functional form among variables of interest and decision-makers (Berrevoets et al., 2024). Despite strong evidence supporting MLCC, diverse stakeholder interests and power dynamics hinder its implementation (Simmelink et al., 2025). This study was to address these challenges by leveraging CDL to evaluate MLCC’s impact on LBW outcomes.

### Related work

Some existing studies are as follows: We used an ML approach to predict the weight range of infants in studies conducted in Belihuloya, Balangoda, Sri Lanka. The study was conducted in the United Arab Emirates (Khan et al., 2022) to estimate infant birth weight and LBW using ML algorithms. The authors conducted their study in Shanghai, China, using an ML approach to estimate fetal birth weight in high-risk pregnancies (Moreira et al., 2019).

Studies in the USA (Lu et al., 2019) investigated fetal weight at varying gestational ages using an ML approach. Studies conducted in Mexico (Campos Trujillo et al., 2020) predict early fetal weight using a support vector machine (SVM). Furthermore, the study conducted in China (Tao et al., 2021) used hybrid data from electronic medical records with the B-ultrasonic examinations of pregnant women to build a predicted birth weight classifier based on extended short-term memory networks.

Previous studies have employed ML and DL algorithms to predict LBW but have not sufficiently addressed causal inference. For instance, research in Iran (Arayeshgari et al., 2023) compared multiple ML models, including decision trees, random forests, artificial neural networks (ANNs), SVM, and logistic regression, to predict LBW risk factors. Similarly, another study in Iran evaluated eight ML and DL algorithms (XGBoost, LightGBM, and K-nearest neighbors) for LBW prediction but focused solely on predictive accuracy rather than causal effects (Alam et al., 2023). In the United States, ML approaches have been applied to LBW prediction. Yet, these studies also lacked rigorous causal analysis, such as estimating ATE or addressing the precision in heterogeneous treatment effect (PEHE) (Jiao et al., 2024).

A critical gap in these studies is their reliance on associative models rather than causal frameworks, which limits their utility for policy and intervention design. Recent advancements in CDL and doubly robust methods (Bayesian additive regression trees for propensity score estimation) have improved bias reduction and PEHE estimation in observational health data (Jiao et al., 2024; Mbogu, 2023). While ML and DL have been applied to neonatal outcomes, most studies focus on associative predictions rather than causal inference. For instance, (Keles and Bagci, 2023) systematically reviewed 106 AI studies in neonatology and found that primary applications included survival analysis and diagnosis, but few addressed causal relationships or heterogeneous treatment effects. This gap highlights the need for methods such as CDL to move beyond correlation and quantify intervention impacts, such as MLCC on LBW. Therefore, this study aimed to establish cause-and-effect relationships using CDL models that reduce bias and estimate heterogeneous treatment effects on LBW in the MLCC intervention.

The novelty of this work lies in its innovative application of CDL models, specifically CFR-WASS and CFR-MMD, to estimate heterogeneous treatment effects of MLCC on LBW outcomes in a low-resource setting. Unlike previous studies focusing solely on predictive accuracy, this research integrates counterfactual analysis with DL to reduce bias and provide interpretable causal insights. The study uniquely combines causal inference with DNNs to improve propensity score estimation, enabling tailored intervention strategies. Additionally, it offers robust metrics such as PEHE, ATE, and individualized treatment effect (ITE), advancing precision in maternal healthcare decision-making.

Propensity Score Matching (PSM) offers valuable insights for improving maternal healthcare by creating comparable groups from observational data. This approach helps clinicians determine which care strategies prove most effective for particular patient populations by matching women with similar medical histories and risk factors. For health policymakers, PSM serves as a powerful tool to assess real-world program impacts, such as evaluating community health worker initiatives by comparing health outcomes between equivalent groups who did and did not receive the MLCC. The method generates practical evidence to expand successful programs and modify less effective ones, particularly important in settings with limited healthcare resources where data-driven decisions. While PSM provides crucial evidence when clinical trials aren’t feasible, our advanced CDL methods overcome PSM’s constraints by modeling intricate relationships in maternal health data, leading to more nuanced policy recommendations and clinical guidelines. Our CDL framework builds on PSM by addressing its limitations, capturing complex relationships to further improve precision in maternal health strategies (Yu and Kang, 2019).

## Methods and participants

### Data sources

A quasi-experimental study was conducted between August 2019 and September 2020 in the North Shoa Zone in the Amhara Regional State of Ethiopia. This region is home to over two million people, with approximately 2,393,877 individuals residing within its boundaries. Among these residents, 1,207,839 are males, and 1,186,038 are females.

There are a total of nine hospitals in the region. One hospital is a referral center specifically equipped to provide comprehensive emergency obstetric care. The North Shoa Zone boasts 95 health centers. These centers serve as essential points of access to primary healthcare services. In addition to hospitals and health centers, the region has 389 health posts. These health posts are strategically distributed across rural and urban areas, ensuring that even remote communities can access basic healthcare services. Overall, the North Shoa Zone’s healthcare infrastructure strives to address its population’s diverse healthcare needs, emphasizing maternal and child health, emergency care, and community-based services.

A total of 1,166 mothers visiting prenatal and antenatal care clinics during the study period were included. Four primary hospitals in the study area, Shoa Robit, Ataye, Mehal Meda, and Alem Ketema Enat Hospital, were randomly selected using a two-stage stratified cluster sampling technique. These hospitals serve both urban and rural populations and provide delivery services. Samples were equally distributed, and participants were selected using systematic random sampling with an interval of two. Shoa Robit and Ataye hospitals were designated as intervention sites offering MLCC, while Mehal Meda and Alem Ketema Enat hospitals served as control sites. Eligible pregnant women were approached and enrolled until the target sample size was achieved.

### Data collection

Midwives recorded participants’ baseline characteristics, including socio-demographics and obstetric, gynecologic, medical, and surgical histories, using a standard tool via face-to-face interviews and maternal antenatal cards. An independent, blinded data collector from the birth registry collected post-birth outcomes. Intervention exposure and continuity of care data were obtained from medical records and postnatal interviews. To avoid the Hawthorne effect, healthcare providers were blinded to outcome data. Eight midwife data collectors and four supervisors underwent a three-day training program for data collection and extraction.

### Eligibility criteria

The study included pregnant women who were less than 24 weeks’ gestational age at their first antenatal care visit, had a singleton pregnancy, and were classified as low obstetric risk. Women with multiple pregnancies, those planning to seek care from a different provider, or those with a history of medical or obstetric complications were excluded from the study.

### Quasi-experimental setup

#### Treatment group (MLCC)

Antenatal care plays a pivotal role in preventing LBW, with the effectiveness often influenced by the model of care provided. MLCC is increasingly recognized for its positive impact on birth outcomes, including a reduced risk of LBW. This model fosters a strong, trusting relationship between a woman and her consistent midwife or small team of midwives throughout pregnancy, birth, and the postnatal period (Moges et al., 2025). This continuity facilitates early and comprehensive risk identification, allowing for prompt interventions such as nutritional counseling, vigilant monitoring for conditions such as pre-eclampsia, and timely referrals for complications, all of which directly mitigate LBW risk (Sandall et al., 2024). Furthermore, MLCC promotes health education and supports physiological pregnancy and birth processes. By avoiding potentially harmful interventions such as routine episiotomies, elective labor inductions without medical indication, or unnecessary cesarean sections, MLCC helps maintain optimal conditions for fetal growth (Mayberry et al., 2017). This approach reduces interruptions to a natural pregnancy, which can negatively affect birth weight. Studies indicate that MLCC protects against preterm birth and LBW, especially for at-risk women, by improving their access to and engagement with community-based care.

#### Control (other professional)

In contrast, antenatal care models led by other professional groups, such as obstetricians or those involving fragmented standard care, while essential for high-risk pregnancies, may sometimes face challenges in optimizing factors related to LBW prevention for all women. Obstetrician-led care is critical for managing complex medical conditions and severe complications that directly threaten fetal growth and contribute to LBW. However, in lower-risk pregnancies, a more medicalized approach might lead to higher intervention rates without a corresponding benefit in LBW reduction compared to MLCC (Voon et al., 2017).

Continuous care is crucial for preventing LBW. When pregnant women see different providers, it breaks down trust and causes inconsistent health advice, making it harder to spot complications early and leading to poor adherence to medical advice. This lack of consistent care can increase the risk of LBW, so clinical practices should focus on building strong, ongoing relationships between women and their healthcare providers (Fernandez Turienzo et al., 2021).

#### Variables in the study

**Figure 1:**
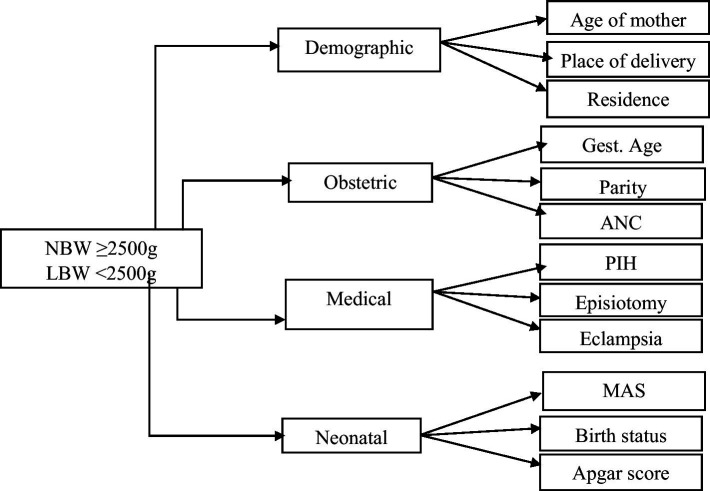
Key factors associated with low birth weight. ANC, antenatal care visit; PIH, pregnancy-induced hypertension; MAS, meconium aspiration syndrome; Gest.Age, gestational age.

This study analyzed binary outcome variables, categorizing newborns into two groups: LBW (≤2,499 grams) and normal birth weight (NBW) (≥2,500 grams). This research framework focused on the causal relationship between MLCC and other professional groups while controlling demographic characteristics, obstetric history, medical factors, and neonatal outcomes influenced LBW risk across these care models (Reza and Salma, 2024), as shown in Figure 1.

### Variable selection

In this study, we initially examined a comprehensive set of over 200 variables encompassing demographic, obstetric, medical, and neonatal factors to investigate their association with LBW, as shown in Figure 1 below. We employed CML techniques combined with feature selection methods to identify the most predictive features while minimizing redundancy and multicollinearity (Moges et al., 2025).

First, we applied univariate analysis to assess the preliminary relevance of each variable. Next, we utilized regularized regression to penalize non-influential predictors, shrinking their coefficients to zero and retaining only the most significant ones. We implemented recursive feature elimination to further refine our selection, which iteratively removes the least important features while optimizing model performance (Moges et al., 2025).

### Causal deep learning algorithms

This study employed TensorFlow 2 and PyTorch to develop CDL models for estimating heterogeneous treatment effects across subgroups. Custom neural networks were designed for specific causal inference tasks, while Scikit-learn handled existing ML. Data processing relied on NumPy and Pandas, with visualizations generated using Matplotlib and Seaborn. Bootstrapping ensured reproducibility, and causal analysis was supported by PyWhy and CausalML.

These models adapt well to non-linear confounding and dynamic factors, addressing the limitations of traditional linear approaches (Pattanayak et al., 2017). The study investigates the use of CDL methods, including DNNs, Counterfactual Convolutional Neural Network (CCNNs), Bayesian Ridge, Bagging Regressor, Treatment Agnostic Representation Network (TARNet), Balancing Neural Networks, CFR-WASS, Causal Effect Variational Autoencoder, and CFR-MMD, to estimate propensity scores in causal inference (Whata and Chimedza, 2022; Ramachandra, 1803).

These advanced CDL models significantly improve treatment effect estimation from real-world clinical data. DNNs and CCNNs excel at analyzing complex patient characteristics for precise treatment comparisons. Bayesian methods incorporate medical expertise into analyses, while ensemble approaches such as Bagging Regressor produce stable results. TARNet and balancing-focused networks (CFR-WASS/MMD) rigorously control for confounding factors in quasi-experimental studies. Most innovatively, the Causal Effect Variational Autoencoder predicts how individual patients would respond to different treatments, enabling truly personalized care recommendations. Together, these methods provide clinicians with more reliable evidence about treatment effectiveness while accounting for real-world data limitations (Keles and Bagci, 2023).

### Counterfactual convolutional neural networks

The PEHE and ATE for CCNNs depend on factors like architecture, training data, and hyperparameters (Kong et al., 2022). While CCNNs excel in image-based tasks, adapting them for causal inference requires careful design. To estimate PEHE and ATE using CCNNs, we have created an architecture that takes covariates and MLCC as input, predicting LBW; this is well-suited for handling sequential data like pregnancy stages and their influence on LBW.

### Bayesian ridge

We have trained our model on the data to estimate PEHE and ATE using Bayesian Ridge, incorporating covariates and MLCC as features. PEHE compares predicted outcomes for MLCC and other professional groups. At the same time, ATE is computed based on average outcomes for MLCC and other professional groups. Therefore, the mathematical expression for Bayesian ridge regression can be represented as follows:


y=Xw+∈

Where: y represents the target variable, X is the design matrix (features), w represents the weight vector (coefficients), and ∈ represents the noise.

### Counterfactual regression with Wasserstein distance

The CFR-WASS improves causal effect estimation by balancing covariate distributions between treatment groups, addressing selection bias, and non-overlapping support (Shalit et al., 2017). Unlike conventional methods like PSM, it minimizes distributional discrepancies using the Wasserstein distance, enhancing accuracy in heterogeneous treatment effects. CFR-WASS excels in quasi-experimental data, particularly for complex relationships and confounding variables, providing reliable Conditional Average Treatment Effect (CATE) estimates. Its robustness makes it especially valuable for evaluating interventions like MLCC compared to standard care models.

### Counterfactual regression with maximum mean discrepancy

Counterfactual Regression (CFR) estimates ITE by predicting potential outcomes under different treatments, such as MLCC. While useful for quasi-experimental data, CFR faces selection bias when comparing MLCC groups to other care providers. CFR-MMD overcomes this by incorporating Maximum Mean Discrepancy to balance covariate distributions in representation space, reducing bias (Shalit et al., 2017). This approach improves ITE accuracy, particularly in complex scenarios such as MLCC evaluation, where covariate balance is crucial for reliable causal inference. CFR-MMD thus strengthens traditional CFR by addressing key limitations in quasi-experimental analysis (Kallus, 2020).

The CFR-MMD improves causal effect estimation by reducing selection bias through distributional alignment. The Gromov-Wasserstein Information Bottleneck framework enhances its precision in evaluating MLCC interventions compared to standard care models (Shi et al., 2019).

### Model training and evaluation

In CDL, rigorous evaluation metrics are essential for validating model performance in estimating treatment effects. The PEHE quantifies accuracy in individual-level effect estimation, with lower values indicating better performance (Lin et al., 2020). The ATE measures bias in population-level effect estimation, where values closer to zero reflect unbiased estimation. Mean Squared Error (MSE) and R-squared (*R*^2^) assess predictive accuracy, though they alone cannot guarantee correct causal identification (Kiriakidou and Diou, 2022). These metrics must be evaluated collectively, as models may achieve strong prediction (high *R*^2^) while failing to recover true causal relationships (high ATE error). Recent methodological work emphasizes the necessity of combining these metrics with robustness checks and out-of-sample validation to ensure strong causal inference, particularly when applying CDL methods to high-stakes domains like clinical decision-making. The optimal model should simultaneously minimize PEHE and ATE error while maintaining reasonable predictive performance (MSE, *R*^2^), with preference given to methods demonstrating stability across different experimental conditions (Huang, 2022).

Beyond these causal-specific metrics, the existing classification measures, accuracy, precision, recall, and area under the curves (AUC), can offer additional insights, particularly when evaluating propensity score models or binary outcomes. Accuracy indicates overall correctness but may be unreliable in imbalanced datasets, such as those with rare treatments. Precision (the proportion of true positives among predicted positives) and recall (the ability to capture all true positives) are especially useful in clinical settings where false treatment recommendations or missed interventions carry significant consequences. AUC evaluates a model’s ability to distinguish between treated and control groups, with higher values suggesting better separation. However, while these metrics help assess model reliability, they do not directly validate causal effects and should always be paired with causal-specific evaluations such as PEHE and ATE. Recent methodological work emphasizes the necessity of combining these metrics with robustness checks and out-of-sample validation to ensure strong causal inference, particularly when applying CDL methods to high-stakes domains, such as clinical decision-making. The optimal model should simultaneously minimize PEHE and ATE error while maintaining reasonable predictive performance (MSE, *R*^2^), with preference given to methods that demonstrate stability across different experimental conditions.

### Learning process of artificial neural networks

Artificial neural networks, particularly multilayer perceptron’s (MLPs), excel at identifying complex patterns in clinical data, such as predicting pregnancy risks or birth outcomes. By analyzing relationships between variables such as maternal health indicators and fetal growth, ANNs can uncover subtle, non-linear associations that existing statistical methods might miss. For clinicians, this means more accurate risk stratification and personalized care plans, for example, flagging high-risk pregnancies for LBW or preterm birth based on nuanced interactions between factors such as pregnancy-induced hypertension (PIH) and ANC adherence (Nahatkar et al., 2025).

**Figure 2:**
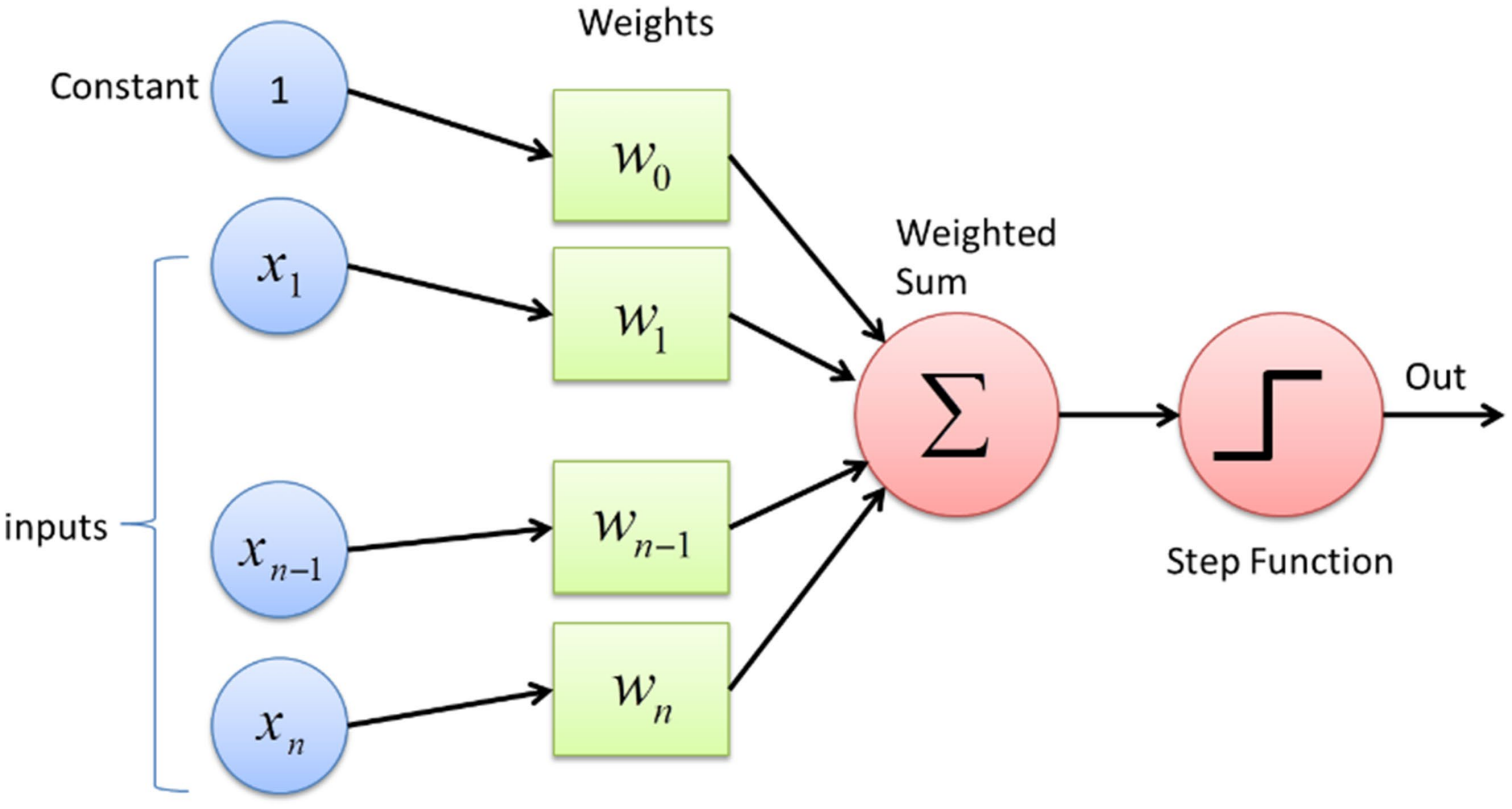
Neural network node diagram (Pang et al., 2020), each node represents a neuron in the figure. The input data flows into the neural network from the input layer. Specifically, the input data values are connected to all neurons in the first layer. Then, each neuron in a layer passes its output to all neurons in the subsequent layer. Finally, the network’s output is on the image’s right side. In regression tasks, this output corresponds to the dependent variable that needs to be estimated.

Figure 2 shows a schematic representation of the mathematical model of an artificial neuron, i.e., a processing element, highlighting input $X_{i}$ , weights ( $w_{0}$ , $w_{1},w_{n-1}$ , and $w_{n}$ ), constant [bias (b) ], ∑ is the summation function, f is the activation function, and Out (y) is the output signal.

This Figure shows how a simple neural network processes medical data to support clinical decisions. Inputs (such as PIH) are assigned importance weights, combined into a weighted sum, and transformed through a step function to produce an output. For clinicians, this mirrors how we intuitively weigh multiple risk factors such as PIH, prenatal death, or meconium aspiration syndrome (MAS) to assess a patient’s risk of complications. The model quantifies this decision-making process, helping to standardize predictions for outcomes such as LBW or preeclampsia based on the combined influence of key clinical variables.

Furthermore, based on the above Figure 2, an ANN is a computational model inspired by the brain’s structure, made up of an input layer, one or more hidden layers, and an output layer (Bland et al., 2020). A DNN is a type of ANN with many hidden layers, allowing it to handle more complex learning tasks. Essentially, all DNNs are ANNs, but not all ANNs are DNNs; only those with multiple hidden layers qualify as DNNs (Goodfellow et al., 2016). In our work, since we use multiple hidden layers, we refer to our model as a DNN rather than a basic ANN.

### Estimating the ATE/CATE, deep learning estimation

Deep learning methods estimate CATE to reveal how MLCC differentially impacts birth outcomes across patient subgroups. ATE quantifies MLCC’s average causal effect on LBW compared to other professionals, guiding targeted maternal interventions (Curth et al., 2024).

It provides an overall estimate of the MLCC effect across the entire population.


$$\mathrm{ATE}=E[Y_{i}(1)-Y_{i}(0)]=E[\tau_{i}]$$

Where $Y_{i}(1)$ and $Y_{i}(0)$ are the potential outcomes, which means NBW and LBW had the unit MLCC or did not receive the treatment, respectively.

The CATE is defined as:


CATE=E[Y(1)−Y(0)∣X=x]

Where: X is the set of selected covariates, and x∈X . The ATE measures an intervention’s overall impact across a population, guiding evidence-based clinical decisions. In contrast, PEHE assesses how accurately models predict ITE responses, enabling personalized care strategies when PEHE values are sufficiently low. Together, these metrics help clinicians balance population-level recommendations with patient-specific interventions (Kent et al., 2018; Ling et al., 2023).

The distinction between ITE and ATE is crucial for clinical interpretation. The ATE represents the average difference in outcomes between a treated group and a control group, providing an overall measure of a treatment’s effectiveness across a population. In clinical practice, the ATE informs decisions about whether a treatment is beneficial on average for a broad patient population, typically guiding policy-level decisions, treatment guidelines for general conditions, and public health interventions (assessing the average impact of allocation of mothers for MLCC and other professionals). However, the ATE can mask significant heterogeneity in treatment response, meaning some individuals may benefit greatly, some may experience no effect, and others may even be harmed. This is where ITE becomes vital. The ITE, on the other hand, quantifies the specific effect of a treatment for a single individual, taking into account their unique characteristics and comorbidities. In clinical practice, ITE is increasingly sought for personalized medicine, enabling clinicians to tailor treatment decisions to individual patients. This approach allows clinicians to predict who is most likely to benefit from a particular therapy, who might experience adverse effects, and who may respond better to alternative treatments (Gopalkrishnan, 2020). Recent advancements in machine learning and causal inference are enabling better estimation of ITEs, facilitating more nuanced clinical decision-making, and optimizing patient outcomes by moving beyond a one-size-fits-all approach to treatment.

## Results

When investigating heterogeneous treatment effects, it is essential to account for confounding variables that may impact both MLCC and LBW. CDL models can integrate these variables, yielding more reliable estimates of the actual MLCC effect.

Several antenatal factors show significant associations with LBW outcomes. Iron/folic acid supplementation demonstrates a protective effect, with unsupplemented mothers showing higher LBW rates (95%, CI: 0.34–0.67, *p* = 0.007). The timing of the first antenatal care visit matters significantly; mothers with delayed initiation have increased LBW risk (*p* = 0.044). PIH emerges as a strong risk factor, with affected mothers having 58% higher LBW rates (7.45% versus 4.72%, *p* = 0.036). While nutrition counseling coverage was high (>94%), the counterintuitive finding of higher LBW among counseled mothers (*p* = 0.0275) warrants further investigation. The allocation of mothers (MLCC compared to other groups) shows a small but significant difference in LBW distribution (*p* = 0.0054). These findings showed that targeted micronutrient supplementation, early antenatal care initiation, and proper management of hypertensive disorders could effectively reduce LBW incidence, as shown in Table 1.

**Table 1:** Association between antenatal care and low birth weight outcomes, in north Shoa zone, Amhara Region, Ethiopia.

Variables	Category	Birth Weight, *n*, %, (95% CI)			
		≥2,500 g	<2,500 g	Chi-square	*p*-value
Maternal Age	< 20 years	86,16.92, (45.3–48.4)	106,16.11, (14.3–16.4)	0.340	0.044
	20–29 years	339,66.73, (55.2–67.4)	437,66.41, (8.3–10.3)		
	≥30 years	83,16.33, (42.1–53.2)	115,17.47, (12.2–15.4)		
Residence	Urban	397, 78.15, (23.3–33.7)	533,81.01, (0.81–0.90)	1.446	0.229
	Rural	111,21.85, (26.3–45.2)	125,18.9, (0.77–0.82)		
Folic acid /Iron	Given	483,95.1,(74.9–78.7)	639,97.12, (1.4–2.8)	3.265	0.0071
	No given	25,4.9, (23.5–28.3)	19,2.88, (0.34–0.67)		
Antenatal Care Visit	First ANC visit	36,7.08, (6.7–7.1)	44,6.68, (2.4–4.5)	4.160	0.044
	2 to 3 visits	209,41.2, (41.2–41.7)	275,41.79, (12.3–14.7)		
	≥4 visit	263,51.77, (51.5–51.7)	339,51.52, (21.3–26.6)		
Nutrition Counseling During Pregnancy	Yes	482, 94.9, (94.9–96.2)	633,96.2, (34.8–42.4)	1.192	0.0275
	No	26,5.12, (3.8–5.2)	25,3.79, (0.34–0.56)		
Allocated group of mothers	Other groups	250,49.22, (49.2–50.8)	334,50.75, (15.2–16.1)	0.275	0.0054
	Midwife-led	258,50.78, (49.3–50.8)	324,49.24, (13.5–15.4)		
Pregnancy-Induced Hypertension	No	484,95.27, (92.6–95.3)	609,92.55, (45.8–58.6)	3.62	0.036
	Yes	24,4.72, (4.7–7.5)	49,7.45, (1.3–1.7)		

The postnatal outcomes reveal several significant associations with LBW. Newborns with MAS showed markedly higher LBW rates [7.3% (0.34–0.78)] versus 3.14% (3.2–7.3), *p* = 0.002, indicating this complication nearly doubles LBW risk. Low Apgar scores (≤7 at 5 min) were significantly more common among LBW infants [27.5% (2.5–3.4)] versus 22.64% (27.5–27.8), *p* = 0.034. Vacuum-assisted deliveries also showed higher LBW prevalence [11.09% (4.6–5.8)] versus 8.27% (8.3–11.1), *p* = 0.045. Postnatal care patterns differed significantly (*p* = 0.03), with LBW infants more likely to receive only one visit (44.8% versus 41.92%). These findings suggest that LBW infants face greater neonatal complications and require more intensive postnatal monitoring, particularly after instrumental deliveries. The increased MAS risk specifically highlights the vulnerability of LBW newborns to birth-related complications (Table 2).

**Table 2:** association between postnatal care and low birth weight outcomes, in north Shoa zone, Amhara Region, Ethiopia.

Variables	Category	Birth weight, *n*, %, (95% CI)			
		≥2,500 g	<2,500 g	Chi-square	*p*-value
Initiation of breastfeeding	After 1 h	121,23.82, (23.8–26.3)	173,26.29, (17.6–18.3)	0.930	0.335
	Within 1 h	387,76.18, (73.7–76.2)	485,73.708, (1.9–2.3)		
Meconium aspiration (newborn health Status)	No	492,96.85, (92.7–96.9)	610,92.7, (73.4–80.5)	9.496	0.002
	Yes	16,3.14, (3.2–7.3)	48,7.3, (0.34–0.78)		
Apgar score ≤7 at 5 min (newborn health Status)	No	393,77.36, (22.6–27.5)	477,72.49, (26.4–29.6)	3.589	0.034
	Yes	115,22.64, (27.5–27.8)	181,27.5, (2.5–3.4)		
Vacuum-assisted delivery	No	466,91.73, (88.9–91.3)	585,88.91, (19.4–20.5)	2.576	0.045
	Yes	42,8.27, (8.3–11.1)	73,11.09,(4.6–5.8)		
Postnatal Care	1 visit	213,41.92, (37.7–46.3)	295,44.8, (41.1–48.6)	1.020	0.03
	2 visit	163,32.08, (28.1–36.3)	200,30.39, (26.9–34.1)		
	3 visit	119,23.43, (19.8–27.3)	148,22.49, (19.3–25.9)		
	4 visit	13,2.55, (1.4–4.3)	15,2.27, (1.3–3.7)		

### Performance metrics of the causal deep learning model for birth weight classification

The DNN model attained a training accuracy of 81.3% and a testing accuracy of 81.4%. When analyzing the dataset composition, NBW conditions accounted for 5.1% of the training and 5.4% of the testing sets. Conversely, LBW constituted 94.9% of the training set and 94.6% of the testing set. Remarkably, the classification accuracy for both normal and LBW conditions exceeded 80% in both datasets. In the training set, 28 NBW cases were correctly classified, resulting in a sensitivity of 86.8%. However, 139 NBW instances were misclassified. For LBW, 636 cases were accurately classified, yielding a specificity of 97.8%. Unfortunately, 14 LBW instances were misclassified. In the testing sample, 8 NBW conditions were accurately classified, achieving 82.9% sensitivity. However, 54 NBW instances were misclassified. For LBW, 276 cases were accurately classified, resulting in a specificity of 96.2%. Regrettably, 11 LBW instances were misclassified. These metrics provide valuable insights into the performance across different birth weight categories in training and testing scenarios presented in Table 3.

**Table 3:** Classification accuracy for low birth weight prediction in training and testing datasets.

Sample	Observed	Predicted		Percent Correct
		No	Yes	
Training	No	28	139	86.8%
	Yes	14	636	97.8%
	Overall Percent	5.1%	94.9%	81.3%
Testing	No	8	54	82.9%
	Yes	11	276	96.2%
	Overall Percent	5.4%	94.6%	81.4%

**Figure 3:**
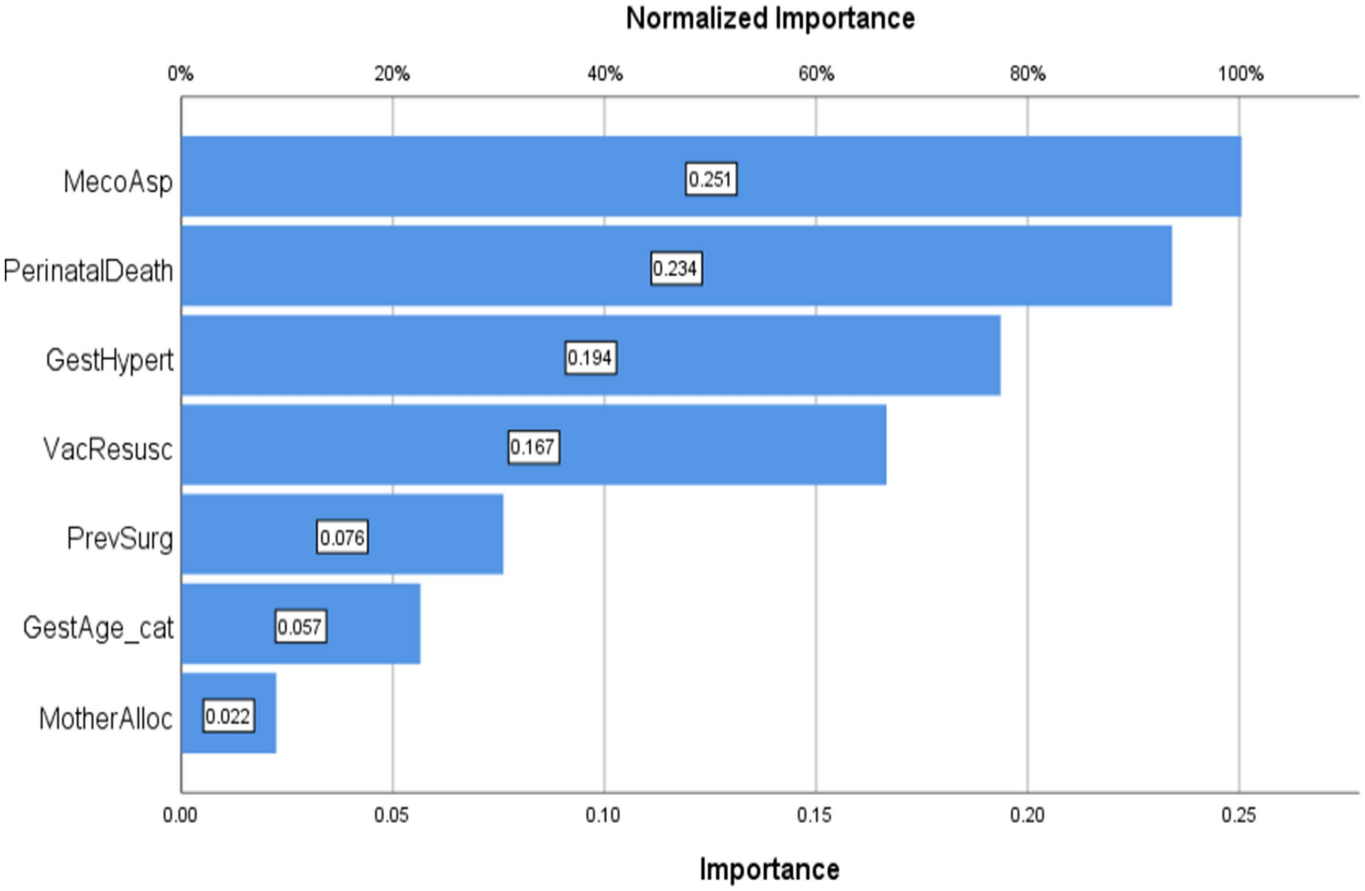
Normalized importance of variables. MecoAsp, meconium aspiration; PerinatalDeath, perinatal death; GestAge_cat, gestational age category; GestHypert, pregnancy-induced hypertension; VacResusc, vacuum baby in need of resuscitation; PrevSurg, previous surgery; MotherAlloc, mother allocation.

Figure 3 presents the normalized importance of key predictors of LBW identified through the feature engineering process analysis. MAS emerged as the strongest predictor, followed by perinatal mortality and gestational age category, with PIH, previous surgery, mother’s allocation, and vacuum-assisted delivery showing progressively lower predictive importance. Our analysis initially identified the top 10 variables linked to LBW. However, after validation with ensemble methods including Random Forest and XGBoost feature importance rankings along with stability selection, only seven key predictors remained consistently significant. These final variables were chosen based on statistical strength, biological relevance, and agreement across multiple causal machine learning models, balancing interpretability and generalizability, as shown in Figure 3. This ranking is associational, not causal, and guides the subsequent causal estimation of ATE, PEHE, and ITE (Moges et al., 2025).

**Figure 4:**
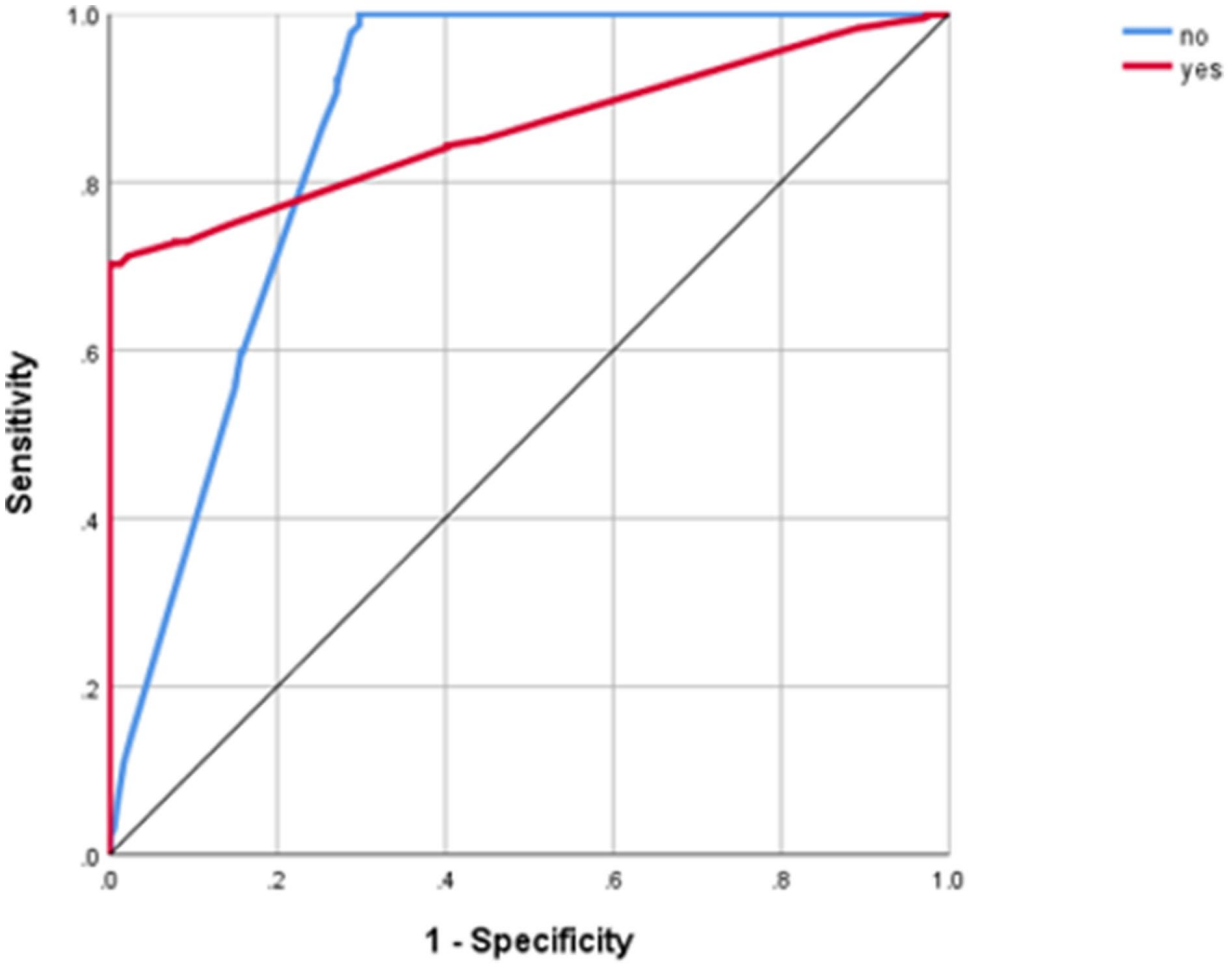
Power of receiver operating characteristic curves.

The receiver operating characteristic (ROC) curves depict the performance of the model for both values of the dependent variable. Notably, all data points lie above the diagonal, signifying effective classification. Furthermore, the AUC, calculated from both the training and testing samples, the AUC quantifies the overall performance of the model; an AUC of 1.0 indicates a perfect model, while an AUC of 0.5 suggests random guessing. Therefore, in this study, the model’s ROC curve yielded an AUC of 0.88, indicating strong classification performance, which indicates better model performance. This substantial AUC underscores the model’s high classification accuracy rate, given in Figure 4.

### Learning mechanism of deep neural networks

**Figure 5:**
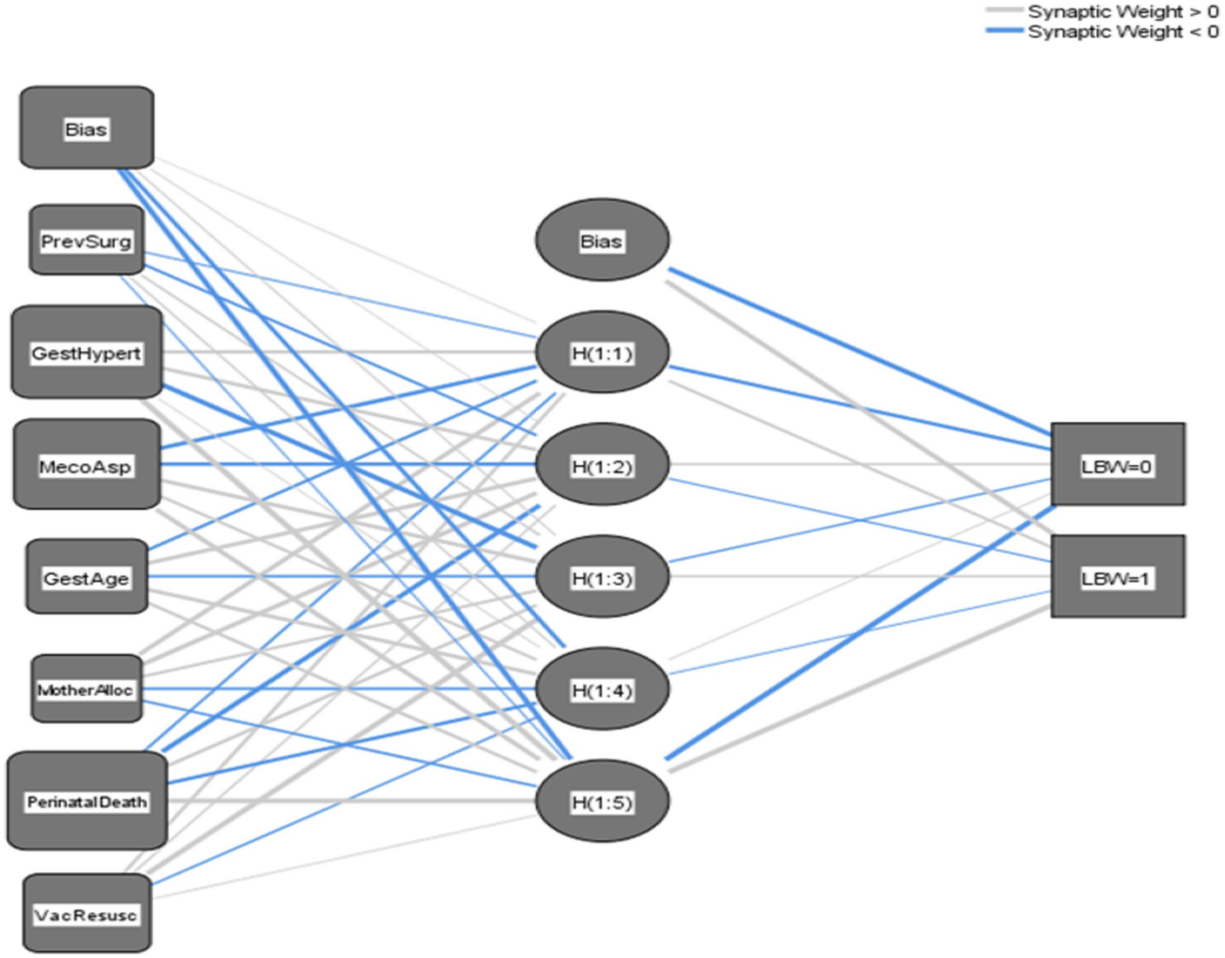
Navigating the neural network unraveling the mysteries of a three-layer multilayer perceptron. MecoAsp, meconium aspiration; PerinatalDeath, perinatal death; GestAge_cat, gestational age category; GestHypert, pregnancy-induced hypertension; VacResusc, vacuum baby in need of resuscitation; PrevSurg, previous surgery; MotherAlloc, mother allocation.

This DNN and MLP model uses seven input variables such as MAS, perinatal death, PIH, vacuum baby in need of resuscitation, gestational age category, mother allocation, and previous surgery to predict two output outcomes (LBW = 0) or (NBW = 1). The model’s single hidden layer, comprising five nodes [H(1:1) to H(1:5)], introduces crucial nonlinearity, allowing it to capture complex relationships within the data that a simple linear model could not. Connections between layers are defined by positive and negative synaptic weights, indicating the influence of each input on hidden layer nodes and, subsequently, the final output, as shown in Figure 5. The study revealed a significant association between MAS and LBW, as evidenced by the neural network’s strong positive weight (0.728) connecting MAS to hidden neuron H(1:2). However, this statistical association does not imply direct causation. MAS typically occurs secondary to fetal distress or other perinatal complications (placental insufficiency or intrauterine hypoxia), which themselves are established risk factors for LBW. The model’s inhibitory weight (−0.345) between perinatal mortality and H(1:3) further suggests complex mediating pathways, where adverse perinatal outcomes may influence birth weight through multiple biological mechanisms rather than through simple direct effects. These findings emphasize that while MAS serves as a clinically useful predictor in the model, it likely represents a marker of underlying pathological processes that independently contribute to restricted fetal growth, rather than functioning as a direct causal agent of LBW. The model’s architecture supports this interpretation, with hidden neurons H(1:1) and H(1:3) showing positive weights (0.165 and 0.166, respectively) for NBW outcomes, while nearly all hidden neurons exhibit negative weights for LBW predictions. So, this captures a nuanced relationship without asserting causal directionality between the observed variables.

Overall, the network architecture transforms input features through hidden layers to predict birth outcomes, with connection weights quantifying each variable’s influence. MAS and gestational age emerge as predictors, demonstrating the model’s ability to identify clinically significant risk factors for LBW.

This interpretation showed the DNN’s ability to capture complex, non-linear relationships between predictors and outcomes (Table 4).


p(y)=(Bias)+(0.310∗input1)−(0.155∗input2)+…+(−0.261)

**Table 4:** Parameter estimates hidden layers and output layers.

Predictor		Predicted						
		Hidden Layer 1					Output Layer	
		H(1:1)	H(1:2)	H(1:3)	H(1:4)	H(1:5)	Normal birth weight	Low birth weight
Input Layer	Bias	0.310	−0.155	0.370	0.093	−0.261		
	Gestational age Category	0.087	−0.022	0.415	0.085	0.471		
	Mother Allocation	0.352	0.072	0.227	−0.281	0.261		
	Perinatal Death	−0.049	−0.029	−0.345	−0.060	−0.181		
	Meconium Aspiration	0.284	0.728	−0.536	0.760	−0.173		
	Pregnancy-induced hypertension	0.144	−0.034	0.249	0.115	−0.002		
	Previous surgery	0.399	−0.045	0.051	0.314	−0.221		
	Vacuum baby in need of resuscitation	0.292	−0.067	0.017	0.056	−0.250		
Hidden Layer 1	Bias						0.152	0.853
	H(1:1)						0.165	−0.150
	H(1:2)						0.004	−0.219
	H(1:3)						0.166	−0.197
	H(1:4)						0.281	−0.193
	H(1:5)						0.278	0.144

**Figure 6:**
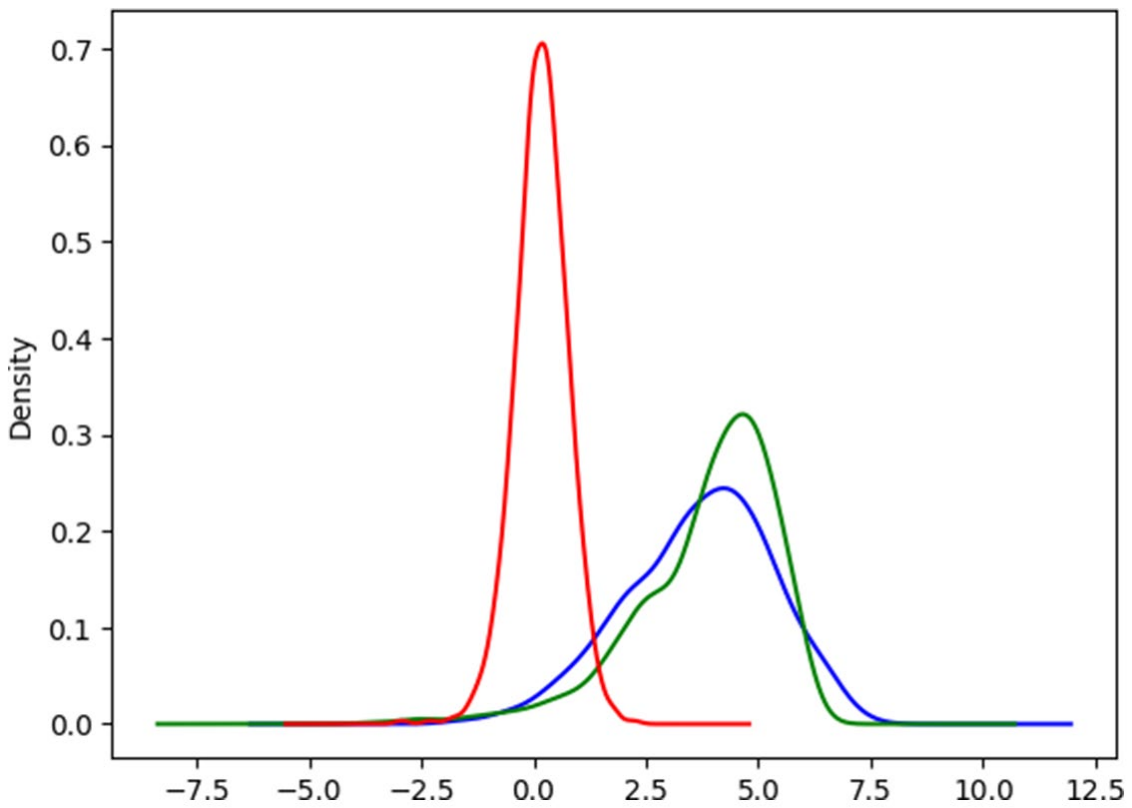
Comparative density plots for CATE estimations. Red indicates the estimated Error CATE, Blue indicates the individualized CATE, and green indicates the individualized CATE True.

Figure 6’s comparative density plot for CATE estimation offers key insights into the impact of MLCC on individual LBW outcomes. The red curve, representing prediction errors, peaks near zero, suggesting the model generally aligns with true effects. However, its spread showed that the challenge of precisely estimating ITEs. The blue curve illustrates the CATE, showing the diverse benefits individuals might gain from MLCC; its broader distribution emphasizes significant variability in individual responses. Ideally, the green curve, depicting the true impact, would reveal a bimodal distribution, indicating distinct subgroups: those who benefit substantially from MLCC and those who experience minimal or no effect. This collective view underscores the necessity of considering heterogeneity and subgroup differences to refine causal inference and tailor MLCC interventions for maximum impact.

### Performance comparison of CDL models for PEHE, MSE, and ATE estimation in LBW analysis

In a comparative analysis of CDL models, CFR-WASS emerges as the top performer, demonstrating superior PEHE (lowest PEHE of 0.35) and strong classification performance across the board (84% accuracy, 82% precision, 85% recall, 0.88 AUC). This robust performance suggests its potential for accurately identifying individuals most likely to benefit from specific medical interventions, thus enabling more personalized treatment strategies. While CFR-MMD also performs well in causal effect estimation (PEHE 0.36, lowest ATE Bias 0.13), CFR-WASS combined strength in both causal and classification metrics makes it particularly promising for clinical applications where both accurate predictions and precise causal inference are critical. For scenarios with moderate-sized datasets where computational efficiency is a priority, Bayesian Ridge Regression and Bayesian Neural Networks offer a practical balance of competitive performance (PEHE 0.42, ATE Bias 0.15, and AUC 0.84–0.86) and significantly faster training times. This makes them viable options for rapid prototyping or deployment in resource-constrained medical environments (Shalit et al., 2017).

Conversely, simpler models such as Lasso Regression show limitations in causal effect estimation (higher ATE Bias 0.23), potentially due to their linear constraints. Hybrid methods, such as Sequential Feature Selection-KNN (PEHE 0.55, AUC 0.75) and Convolutional-KNN (PEHE 0.48, AUC 0.80), generally underperformed, indicating that their presumed advantages for smaller sample sizes might not hold in this context. The notable poor performance of Generative Adversarial Net for Individualized Treatment Effects (PEHE 0.60, ATE Bias 0.30, and AUC 0.71) is attributed to training instability and data inefficiency with the dataset size of 1,166, showing their unsuitability for direct causal inference in such settings. The consistent minimal precision-recall gaps among top-performing models further underscore their balanced predictive capabilities, which are vital for reliable medical decision support, as shown in Table 5.

**Table 5:** Evaluation of causal deep learning models (*n* = 1,166, 80% training, 20% testing).

Methods	PEHE ↓	ATE Bias ↓	MSE ↓	*R*^2^ ↑	Training TIME	Accuracy	Precision	Recall	AUC
Counterfactual Convolutional Neural Network	0.43	0.17	1.11	0.81	Very slow	0.78	0.75	0.80	0.82
Bayesian Ridge Regression	0.42	0.15	1.08	0.82	Fast	0.80	0.77	0.82	0.84
Lasso Regression	0.42	0.23	1.14	0.75	Slow	0.76	0.73	0.78	0.79
1. Convolutional Neural Network-K-Nearest Neighbors	0.48	0.20	1.15	0.78	Fast	0.77	0.74	0.79	0.80
2. Sequential Feature Selection-K-Nearest Neighbors	0.55	0.26	1.30	0.72	Slow	0.72	0.70	0.74	0.75
3. Bagging Regressor	0.45	0.18	1.12	0.80	Moderate	0.79	0.76	0.81	0.83
4. Treatment Agnostic Representation Network	0.39	0.18	1.16		Moderate	0.80	0.78	0.82	0.75
5. Bayesian Neural Network	0.42	0.15	1.08	0.81	Slow	0.81	0.78	0.83	0.86
6. Counterfactual Regression-Wasserstein Distance	0.34	0.11	1.02	0.83	Moderate	0.84	0.82	0.85	0.88
7. Conditional Variational Autoencoder	0.52	0.24	1.25		Moderate	0.74	0.72	0.76	0.77
8. Generative Adversarial Net for Individualized Treatment Effects	0.60	0.30	1.40	0.68	Extreme	0.68	0.65	0.70	0.71
9. Counterfactual Regression-Maximum Mean Discrepancy	0.36	0.13	1.16		Moderate	0.83	0.81	0.84	0.87
Deep neural network	0.37	0.13	1.04		Slow	0.82	0.80	0.83	0.87

↓ = shows decrease in value, ↑ = shows increase in value.

Given the evaluation metrics in Table 5, the next step involves estimating the causal effects of MLCC on LBW compared to other care models. This will entail quantifying the PEHE to gauge individual-level causal accuracy, the ATE error to assess population-level bias, and the ITE for personalized causal insights for each mother. Additionally, we will incorporate classification measures such as accuracy, precision, recall, and AUC to further evaluate the model’s performance.

For clinicians, CFR-WASS, the $\sqrt{\in_{\text{PEHE}}}$ , and the ATE measures are vital for effective decision-making. A low PEHE of 1.006 ± 0.03 signifies the model’s excellent ability to predict ITE, allowing for highly personalized interventions. Conversely, a low ATE of 0.24 ± 0.21 with high variability suggests that while there might be a small average benefit across a population, the effect on individual patients can differ significantly. Therefore, prioritizing a low PEHE is crucial for clinicians to tailor interventions precisely to each patient, moving beyond generalized averages to truly optimize care. Therefore, the proposed CFR-WASS achieved good performance and outperformed state-of-the-art models, and the model tuning or ensemble methods may enhance performance (Shi et al., 2019).

The CFR-MMD’s precise estimates (ITE 0.34 ± 0.12, ATE 0.24 ± 0.01) help clinicians identify which mothers would benefit most from interventions while assessing overall treatment impact. Though results are consistent, real-world validation remains important. This approach enables targeted care for high-risk pregnancies while guiding population-level decisions about resource allocation.

CEVAE’s higher $\sqrt{\in_{\text{PEHE}}}$ (3.21 ± 0.32) and ATE (1.23 ± 0.23) indicate broader variability in its predictions, suggesting better capture of individual patient differences but with less precision. While this helps identify nuanced treatment responses, the wider ranges mean clinical decisions should be cautious, prioritizing high-risk cases where personalized benefits outweigh uncertainty.

The CCNN model shows moderate precision ( $\sqrt{\in_{\text{PEHE}}}$ 2.25 ± 0.25, ATE 2.65 ± 1.45), making it suitable for identifying general treatment trends but requiring cautious interpretation for individual cases. In contrast, Bayesian Ridge delivers more reliable estimates (PEHE 1.12 ± 0.021, ATE 1.32 ± 0.75), supporting both personalized and population-level decisions. Clinically, this means Bayesian Ridge is better suited for guiding interventions, while CCNN may help screen broader risk patterns, though both benefit from further refinement for high-stakes prenatal care.

These findings indicate that our proposed model can extract deep, representative, and discriminative features related to the assigned MLCC, leading to improved ITE estimation performance. The Bayesian ridge stands out due to its high PEHE and reasonable ATE estimation. The bagging regressor performs well in ATE estimation but has moderate precision. Consider the trade-offs between PEHE and ATE estimation when choosing the best method for your specific use case.

In conclusion, the proposed CFR-WASS and CFR-MMD models demonstrated superior performance in terms of $\sqrt{\in_{\text{PEHE}}}$ , outperforming state-of-the-art models in estimating causal effects for LBW outcomes. Based on their CFR-WASS and CFR-MMD outperform other CDL models in estimating PEHE, ATE, and ITE, as evidenced by lower Mean ± Standard Error values in comparisons (Table 6).

**Table 6:** Causal deep learning algorithm for estimating (mean ± standard error) on maternal and neonatal dataset.

Methods	$\sqrt{\in_{\text{PEHE}}}$	$\in_{\mathrm{ITE}}$	$\in_{\mathrm{ATE}}$
Counterfactual Convolutional Neural Network	2.25 ± 0.25	2.15 ± 1.23	2.65 ± 1.45
Bayesian Ridge Regression	**1.12** ± **0.021**	2.45 ± 0.75	1.32 ± 0.75
Lasso Regression	6.65 ± 0.45	5.25 ± 0.43	0.92 ± 0.06
Convolutional Neural Network-K-Nearest Neighbors	3.54 ± 0.52	2.4 ± 0.3	0.85 ± 0.35
Sequential Feature Selection-K-Nearest Neighbors	2.9 ± 0.41	2.6 ± 0.02	0.75 ± 0.25
Bagging Regressor	5.35 ± 1.47	4.14 ± 0.27	5.35 ± 1.32
Treatment Agnostic Representation Network	**1.23** ± **0.52**	1.53 ± 0.49	0.65 ± 0.16
Bayesian Neural Network	1.21 ± 0.12	1.09 ± 0.13	0.59 ± 0.01
Counterfactual Regression-Wasserstein Distance	**1.006** ± **0.03**	**0.25** ± **0.01**	**0.24** ± **0.21**
Conditional Variational Autoencoder	3.21 ± 0.32	2.15 ± 0.64	1.23 ± 0.23
Generative Adversarial Net for Individualized Treatment Effects	2.44 ± 0.08	1.24 ± 0.56	0.67 ± 0.14
Counterfactual Regression-Maximum Mean Discrepancy	**1.012** ± **0.001**	0.34 ± 0.12	0.45 ± 0.01
Deep Neural Network	**1.45** ± **0.051**	2.3 ± 0.32	0.78 ± 0.63

Bold values indicate the algorithm with the best performance (lowest mean) for each causal estimation metric. The mean shows the performance, while the standard error reflects its precision.

A feed-forward deep neural network (FFDNN) facilitates causal inference by modeling intricate, non-linear relationships between treatment, such as MLCC, and LBW, all while accounting for confounding factors. The network’s structure allows it to generate counterfactual predictions by estimating potential outcomes for each observation under both MLCC and other professional scenarios. Through its internal layers, the FFDNN can identify varied treatment effects across different groups, offering predictions for both ITE and ATE effects. Its ability to approximate complex functions helps address the core challenge of causal inference: that only one potential outcome is ever observed for a given subject. Careful regularization and architectural design are crucial for the FFDNN to yield dependable causal predictions, rather than just correlational associations.

To solve a binary classification problem, we combine sigmoid output units with maximum likelihood. A sigmoid output unit has 2 components; one is which uses a linear layer to compute.

z=w∗h+b, and then it uses an activation function to convert z into a probability


$$Z_{1}=w_{11}+w_{12}+w_{13}+w_{14}+w_{15}+w_{16}+w_{17}+W_{0}+\theta_{1}$$

$$\begin{array}{l} Z_{1}=-0.738+0.568+(-0.245)+(-0.8149)+(-0.897)+(-0.581)\\ +0.1489+0.99+0.156=-1.413 \end{array}$$

Now the sigmoid function


$$f(\text{inpu}t_{1})=\frac{1}{1+e^{-(\text{inpu}t_{1})}}$$

$f(z)=\frac{1}{1+e^{-(-1.413)}\;}=0.1956$ , the predicted value of LBW


$$Z_{2}=w_{11}+w_{22}+w_{33}+w_{44}+w_{55}+w_{66}+w_{77}+\theta_{2}$$

$$\begin{array}{l} Z_{2}=0.1229+(-0.1538)+(-0.1694)+0.3538+0.3494+0.34939\\ +(-0.3857)+0.556+0.9875=2.01009. \end{array}$$

$$f(\text{inpu}t_{2})=\frac{1}{1+e^{-(\text{inpu}t_{2})}}$$

**Figure 7:**
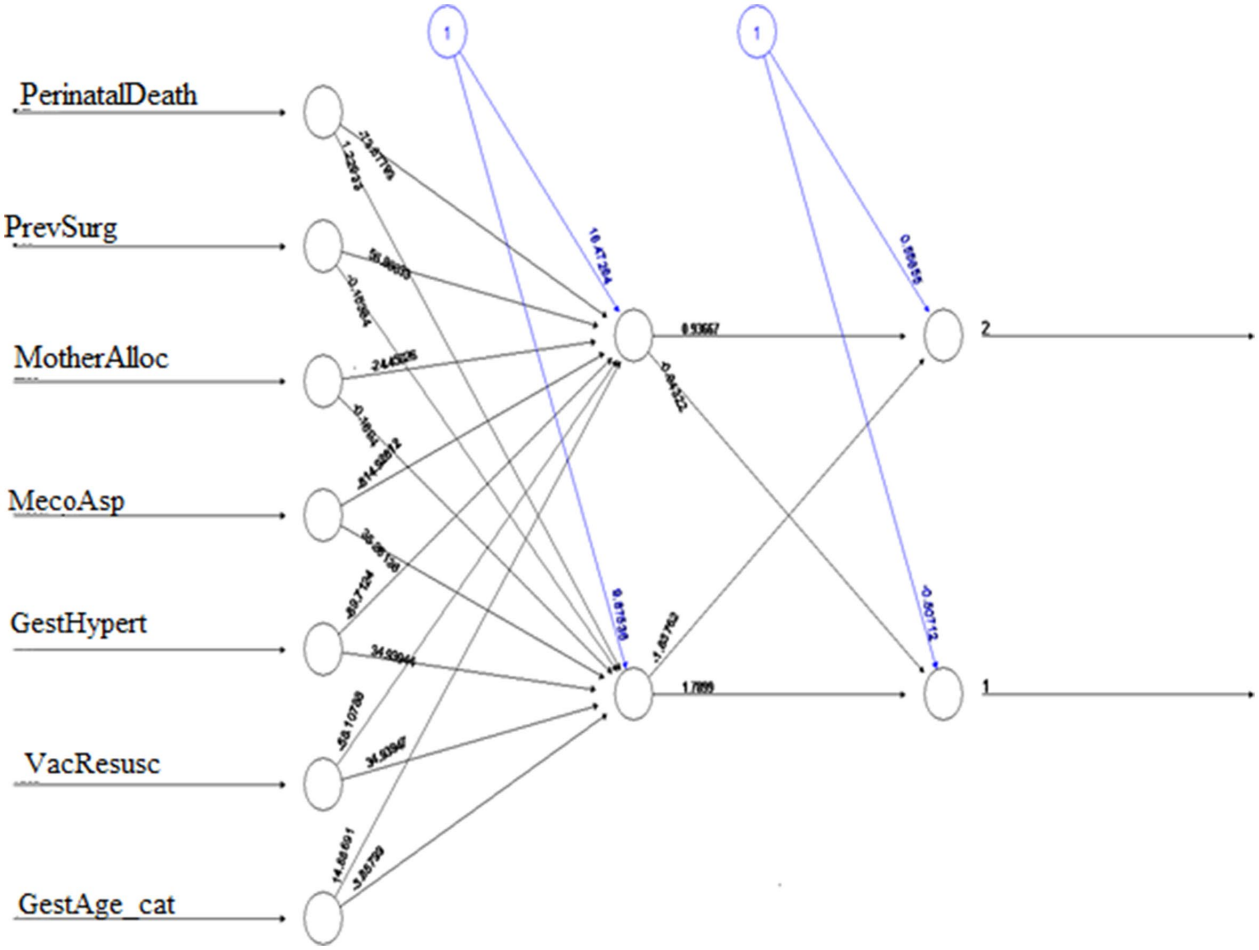
Feed-forward deep neural network. MecoAsp, meconium aspiration; PerinatalDeath perinatal death; GestAge_cat, gestational age category; GestHypert, pregnancy-induced hypertension; VacResusc, vacuum baby in need of resuscitation; PrevSurg, previous surgery; MotherAlloc, mother allocation.

The logistics function $\;f(z)=\frac{1}{1+e^{-2.01009}\;}=0.882$ , predicts an 88.2% probability of NBW when mothers receive MLCC, well above the 0.5 classification threshold. This demonstrates MLCC’s strong protective effect against LBW, as the model consistently associates MLCC adherence with higher probabilities of normal birth outcomes. The results quantitatively confirm that structured ANC significantly reduces LBW risk, showing the MLCC’s clinical importance. These findings underscore the need to expand MLCC access to improve neonatal health outcomes, as shown in Figure 7.

## Discussion

Low birth weight babies face health complications, but not all require interventions (Chen et al., 2013); the impact of LBW was associated with infant mortality and long-term health issues. The prediction of LBW at birth is based on the analysis of different characteristics of newborn babies and mothers. Some characteristics classified as the most important features include MAS, perinatal death, PIH, vacuum babies in need of resuscitation, gestational age category, mother allocation, and previous surgery. This study indicates that higher maternal weight was associated with larger birth weights in babies, which is consistent with (Alabbad et al., 2024). Our study found significant differences in folic acid/iron supplementation (97.1% versus 95.1%, *p* = 0.007) and PIH (7.5% versus 4.7%, *p* = 0.036) between LBW and normal birth weight groups. These findings align with current evidence showing iron-folate supplementation reduces LBW risk by 19% (0.81, 0.71–0.93) (Zenebe et al., 2021), while PIH increases LBW risk 2–3 fold (Yang et al., 2022). These preventable risk factors appear to play a significant role in low birth weight outcomes, underscoring the need for enhanced prenatal care strategies.

Our findings, supported by CDL feature selection methods, identified MAS as the strongest predictive factor for adverse neonatal outcomes compared to other features (Moges et al., 2025). However, while MAS is highly associated with complications in LBW infants, it does not causally precede LBW. Instead, existing literature suggests that MAS is a consequence of intrapartum events rather than a direct cause of LBW. The MAS was a significant cause of respiratory distress in newborns, occurring in about 10–15% of infants born through meconium-stained amniotic fluid (Uniyal et al., 2021). Studies have shown that MAS is more common in term babies and those with LBW (Uniyal et al., 2021). However, it can also affect post-mature and small-for-date infants and those weighing over 2,500 g (Jain et al., 2020). Critically, recent research showed that when MAS develops in infants with LBW, it is associated with a significantly increased risk of severe outcomes and mortality. Although LBW does not cause meconium passage, the physiological vulnerabilities of LBW infants, such as immature lungs, compromise their ability to cope with MAS, leading to a higher incidence of complications such as birth asphyxia, hypoxic–ischemic encephalopathy, seizures, septicemia, and persistent pulmonary hypertension (Jain et al., 2020). Consequently, MAS substantially contributes to neonatal morbidity and mortality, with birth asphyxia being a common cause of death, particularly in vulnerable groups such as LBW infants. The WHO underscores the importance of universal, high-quality perinatal care, including vigilant monitoring and appropriate resuscitation for all newborns, irrespective of gestational age or birth weight, to mitigate adverse neonatal outcomes and address the heightened risks faced by LBW infants with MAS. Early diagnosis and prompt treatment are crucial for improving outcomes (Uniyal et al., 2021; Widiyaningrum et al., 2020).

The findings of this study align with recent research that showed the importance of maternal and neonatal interventions in reducing LBW. The significant association between folic acid/iron supplementation and reduced LBW (*p* = 0.0071) is consistent with studies demonstrating the critical role of micronutrients in improving birth outcomes (Hunter et al., 2023; Johnson, 2022). Similarly, the protective effect of MLCC (*p* = 0.0054) corroborates evidence from recent trials showing that continuity of care models, particularly MLCC approaches, significantly reduce adverse neonatal outcomes (Fikre et al., 2023; Mose et al., 2023). The association between adequate ANC visits and reduced LBW (*p* = 0.044) further supports global recommendations emphasizing the importance of regular ANC visits in improving maternal and neonatal health (World Health Organization, 2021; Dandona et al., 2022).

The findings of this study indicate that ANC visits have a significant effect on the risk of LBW, which contrasts with a previous study conducted in Adwa General Hospital, Northern Ethiopia, which reported no significant association between ANC attendance and LBW among term newborns (Gebregzabiherher et al., 2017; Hailu and Kebede, 2018). This discrepancy may be attributed to differences in study populations, quality of ANC, or healthcare infrastructure. While our results align with global evidence emphasizing the protective role of ANC in reducing LBW (Neupane et al., 2023; Katiso et al., 2020; Wachamo et al., 2019), further context-specific research is needed to explore these variations and optimize maternal care strategies.

Similarly, a 2022 systematic review published in Frontiers in Public Health demonstrated that increased ANC utilization was associated with a 20–30% reduction in LBW incidence, particularly in low- and middle-income countries where access to quality ANC remains a challenge (Engdaw et al., 2023). Another study in (Kassaw et al., 2023) emphasized that ANC visits facilitate early detection and management of conditions such as hypertension and anemia, which are known contributors to LBW. Furthermore, a 2020 WHO multi-country analysis reiterated that at least eight ANC contacts, as per the updated WHO guidelines, further reduce LBW risks by ensuring continuous maternal health monitoring and interventions. The findings highlight that ANC visits are a key modifiable factor in reducing LBW. These results align with existing research and support the need for stronger ANC policies and programs worldwide.

The identification of PIH (*p* = 0.036) and MAS (*p* = 0.002) as significant risk factors for LBW aligns with existing literature, which highlights their detrimental impact on fetal growth and neonatal outcomes (Gomez-Lumbreras et al., 2024; Adugna et al., 2025). However, unlike some prior studies, this study did not find a significant association between ANC attendance and LBW (*p* > 0.05), contrasting with evidence suggesting that adequate ANC reduces LBW risk through early detection and management of complications such as PIH (Tekeba et al., 2024). Additionally, while some studies report protective effects of urban residence and timely breastfeeding initiation against LBW, our findings showed no significant associations (residence: *p* = 0.229; breastfeeding initiation: *p* = 0.335). These discrepancies may stem from variations in healthcare access, ANC quality, or population characteristics, underscoring the need for context-specific interventions to optimize maternal and neonatal health outcomes (Basile Ibrahim et al., 2022; D’Hollander et al., 2025). In this study, we conducted a methodology scoping review, which identified DL causal predictive modeling for MLCC, with the main differences between the methods being the source of data from which the causal effects are estimated. We identified that when the causal effects required for the predictions were fully estimated from the quasi-experimental data, methods were available for predictions under MLCC. We developed a guide for the predictive analysis of PEHE in a quasi-experimental study. Predictive precision of heterogeneity treatment analysis aims at MLCC effects (Lin et al., 2021).

Significant efforts have recently been made to utilize ML techniques for causal inference problems. One notable application is estimating heterogeneous treatment effects. These efforts aim to enhance our understanding and improve outcomes in various domains (Athey and Imbens, 2016), propensity score modeling, and neighbor matching for ITE. DL, a subset of AI, is crucial in estimating MLCC effects (Ren et al., 2023; Davidson and Boland, 2021).

The findings align with (Simmelink et al., 2025), who showed the role of leadership and collaborative efforts in successfully implementing MLCC. Our results further demonstrate that MLCC, when supported by robust policies and interdisciplinary collaboration, can significantly reduce adverse neonatal outcomes such as LBW.

The DNN has been employed to estimate heterogeneous treatment effects within the causal inference framework. DL’s ability to handle complex confounding factors is valuable for understanding LBW and enhancing outcomes (Koch et al., 2025). In this paper, we built a DNN classifier, Propensity Net, for propensity score-based matching to estimate ITE and ATE (Ramachandra, 1803).

The results show that CFR-WASS achieves the best performance with the highest accuracy (84%), precision (82%), recall (85%), and AUC (0.88), along with the lowest PEHE (0.34) and ATE bias (0.11). This aligns with recent findings by (Shalit et al., 2017), who demonstrated that Wasserstein-based methods excel in causal inference by effectively balancing covariate distributions. Similarly, the strong performance of CFR-MMD (AUC = 0.87) supports (Håkansson et al., 2020) work on distribution matching for unbiased treatment effect estimation. In contrast, simpler models such as Lasso Regression (AUC = 0.79) and Generative Adversarial Net (AUC = 0.71) underperform, consistent with (Koch et al., 2024), who showed their limitations in handling complex causal relationships. These findings reinforce the superiority of advanced CDL methods in precision medicine applications. The high AUC scores(≥0.86) for top models validate their discriminative power, supporting their use in precision healthcare applications.

The DNN model’s performance (81.3% accuracy) aligns with findings from (Keles and Bagci, 2023), who reported that DL models in neonatology achieve high accuracy (95% for Retinopathy of Prematurity diagnosis) but often lack interpretability. Our use of neural network weight analysis (e.g., H(1:2) for MAS) addresses this limitation by providing clinically actionable insights, a direction recommended for future AI applications in neonatal care (Keles and Bagci, 2023).

In a DNN, parameter estimates serve as independent variables. These estimates typically correspond to weights and biases associated with neuron connections. In DNN architecture, a single hidden layer is utilized. Models with additional layers did not perform well (Montesinos López et al., 2022; Hassoun, 1995). Hidden layers play a crucial role in capturing non-linear patterns within the data. Without hidden layers, the DNN behaves similarly to a linear regression model, unable to detect nonlinearity (Hussain et al., 2019). In our chosen model, the hidden layer consists of five nodes (neurons). Each node represents a specific combination of input features. The DNN exhibits nonlinearity because the effects at each node vary. Some independent variables have positive effects for one set of observations while having adverse effects for another set. This dynamic behavior results in mean scores near zero, reflecting the intricate interplay of variables (Alzubaidi et al., 2021).

The MLCC model demonstrates better clinical outcomes than standard care, with lower rates of medical interventions (epidurals, forceps delivery, episiotomies) and higher rates of natural births and patient satisfaction (Sandall et al., 2016). Patient outcomes under MLCC were assessed by clinicians using standardized protocols, ensuring reliable validation. A structured medical records system was essential for tracking care continuity, enabling consistent evaluations and data-driven improvements in maternal and neonatal health (Hailemeskel et al., 2022).

## Strengths, limitations, and future work

This study contributes to the growing body of literature on causal inference using DNN. Its primary strength lies in addressing causal inference through the potential outcome framework, building and optimizing custom DL models for causal estimation, and adapting these models to predict PEHE effects on LBW. DL models offer significant advantages, such as automatically extracting relevant features from data, reducing the need for manual feature selection, and effectively capturing non-linear relationships. However, the study focuses on quasi-experimental designs, which inherently face limitations due to confounding and uncontrolled variables. While DL models excel in handling complex data, they struggle with complex confounding structures and often lack interpretability, making it challenging to understand the underlying causal mechanisms.

Quasi-experimental designs are particularly prone to selection bias due to non-random assignment, leading to imbalances between MLCC and other professional groups and potentially compromising the validity of results. Additionally, unmeasured confounders can obscure causal relationships, further complicating accurate effect estimation. To address these challenges, we recommend future research to explore Double/Debiased Machine Learning (DML), a state-of-the-art algorithm that provides unbiased, root-n-consistent estimators for ATE, heterogeneous treatment effects, and their confidence intervals. DML enhances adjustments for non-linear confounding relationships, offering a more robust approach to causal inference in complex datasets. By integrating DML, future studies can improve the accuracy and reliability of causal estimates, advancing the intersection of DL and causal inference in healthcare and beyond.

## Conclusion

In this study, we employed DL causal inference techniques, such as CCNN, CFR-WASS, causal effect variational autoencoder, and balancing neural network, for measuring the effectiveness of PEHE, ITE, and ATE of LBW predictions for capturing more complex patterns and relationships of the given data. The analysis revealed that MAS was the strongest predictor, but other factors such as gestational age and perinatal mortality also played a role.

In this study, the DNN model delivered reliable results, reaching 81.3% accuracy on the training set and 81.4% on the test set, indicating stable predictive performance. Its impressive AUC score of 0.88 further validates its ability to accurately predict LBW. The analysis of the hidden layer identified H(1:1) for the allocation of the mother, H(1:2) for the allocation of the mother, H(1:3) for perinatal death, and H(1:3) for MAS with positive and negative influences on LBW, respectively. Therefore, this indicated that, hidden layer provided insights into the specific influences of various factors on LBW.

CFR-WASS outperformed all other models, achieving the highest accuracy (84%), precision (82%), recall (85%), and AUC (0.88). It also had the lowest errors, with a PEHE of 0.34 and ATE bias of 0.11. In predicting LBW, CFR-WASS maintained strong performance with a PEHE of 1.006 and an ATE of 0.24, surpassing competing methods. Both CFR-WASS and CFR-MMD effectively estimated causal effects, showing that their potential to enhance maternal and neonatal healthcare interventions, particularly by evaluating the impact of ANC visits on LBW risk. In Addition, the FFDNN model, using a sigmoid function, predicted a higher probability of 0.882 of NBW for newborns whose mothers followed MLCC compared to a lower probability of 0.1956 for LBW, reinforcing the importance of adequate ANC in improving birth outcomes. These findings showed that a critical role of MLCC in reducing LBW, particularly in resource-limited settings. By ensuring consistent antenatal monitoring, timely folic acid/iron supplementation, and adherence to ANC visits, MLCC models demonstrate promise in improving maternal and neonatal outcomes. Future research should prioritize cost-effectiveness analyses and implementation strategies to scale MLCC programs, informing policies aimed at LBW prevention and maternal–infant health equity.

## Data Availability

The raw data supporting the conclusions of this article will be made available by the authors, without undue reservation.
